# The Remarkable Plasticity of Macrophages: A Chance to Fight Cancer

**DOI:** 10.3389/fimmu.2019.01563

**Published:** 2019-07-12

**Authors:** Nadège Bercovici, Marion V. Guérin, Alain Trautmann, Emmanuel Donnadieu

**Affiliations:** ^1^INSERM, U1016, Institut Cochin, Paris, France; ^2^CNRS, UMR8104, Paris, France; ^3^Université Paris Descartes, Sorbonne Paris Cité, Paris, France

**Keywords:** cancer, macrophages, T cells, immunotherapy, migration, inflammation

## Abstract

It is well established that tumor-associated macrophages (TAM) found in most advanced tumors have a pro-tumoral role. In this context, TAM limit the activity of tumor-infiltrating lymphocytes (TIL), and a number of mechanisms have been described including a trapping in the stroma, impeding TIL to reach malignant cells. Based on these results, a number of therapeutic approaches have been designed to deplete TAM. However, during tumor regression induced by immunotherapeutic treatments, recent studies revealed that TAM can switch from pro-tumoral to anti-tumoral and actively cooperate with TIL. Here, we will review the two faces of TAM in their interaction with TIL. We will summarize how they can inhibit T cell activities in growing tumors, and how they may also, together with T cells, successfully contribute to tumor eradication after an appropriate stimulation. Finally, we will discuss current promising therapies combining TAM reprogramming with T cell-based immunotherapy.

## Introduction

Macrophages are amongst the most versatile cells in the body. Resident macrophages are abundant in all tissues where, like microglia in the brain or Kuppfer cells in the liver, these “*pro-tissular macrophages*” contribute to optimize the functioning of the tissue in which they are, by maintaining it clean and preventing an unnecessary inflammation (1). Besides, following an appropriate stimulation, e.g., following an infection, macrophages may be key contributors to immune responses (2). They participate to a variety of functions, primarily as effector cells to eliminate the invading bodies but also to drive an acute inflammation, to promote the recruitment of other immune cells as well as to present antigens to T cells. The switch from the pro-tissular, anti-inflammatory state to the pro-immune, inflammatory one, may take place within a few minutes. This is what happens to subcapsular macrophages when they detect the arrival of pathogens in the lymph node subcapsular sinus (3). This switch may take hours or days, when it involves the recruitment of blood monocytes, followed by their appropriate differentiation in the tissue. Even though the distinction between *pro-tissular* and *pro-immune* macrophages shares similarities with the M2/M1 distinction, we favor the idea that the most important difference between these two macrophage subtypes is functional rather than phenotypic.

In advanced tumors, macrophages favor tumor growth and are associated with a bad outcome in most cancers. Therefore, tumor-associated macrophages (TAM) are usually considered as simply “pro-tumoral”. This has not always been the case. In the 1990s, a potential role of macrophages for cancer treatment has been a popular idea and this concept has begun to emerge. Indeed, in sensitized tumors, macrophages may be anti-tumoral, with the modulation of some gene expression (4).

**Figure 1:**
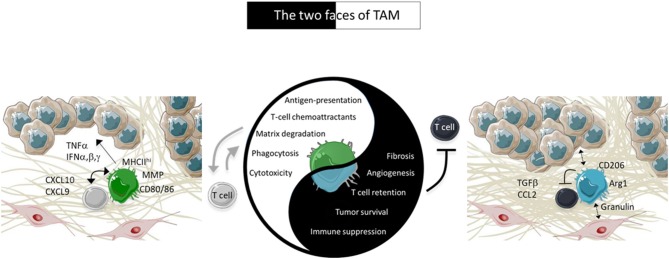
Characteristics of TAM in tumors. In advanced tumors, TAM are pro-tissular and promote tumor growth by several ways including by trapping TIL in the stroma. However, under appropriate activation, TAM positively cooperate with TIL to induce tumor regression. The pro-tissular and pro-immune TAM subtypes are present in two different environments depicted in the figure. Pro-tissular TAM reside in a mesenchymal environment enriched in a dense ECM network and TGFβ. Conversely, pro-immune TAM are distributed in an inflammatory milieu enriched in type I IFN and T-cell chemoattractants.

We will summarize here some specific consequences of the functioning of macrophages in progressing tumors, in which their dominant role is pro-tumoral and immunosuppressive. In particular, we will focus on the mechanisms by which TAM limit TIL from reaching tumor cells. We will continue by considering how one can favor the switch of TAM to pro-immune cells exerting an anti-tumoral action. For these two TAM faces, our focus will be on positive or negative interactions between TAM and TIL, as summarized in the Figure 1.

## TAM Inhibit T Cell Activities in Progressing Tumors

TAM can promote tumor growth by a variety of mechanisms that include tumor cell proliferation, metastasis, angiogenesis and inhibition of T cell anti-tumoral activities. A considerable number of excellent reviews have been published on the various ways in which TAM contribute to tumor growth [for instance see (5)]. Yet, the mechanisms by which TAM negatively control T cells are not completely understood and we would like to focus on those related to intratumoral T cell migration.

### TAM Impair T Cell Migration Within Tumors

**Figure 2:**
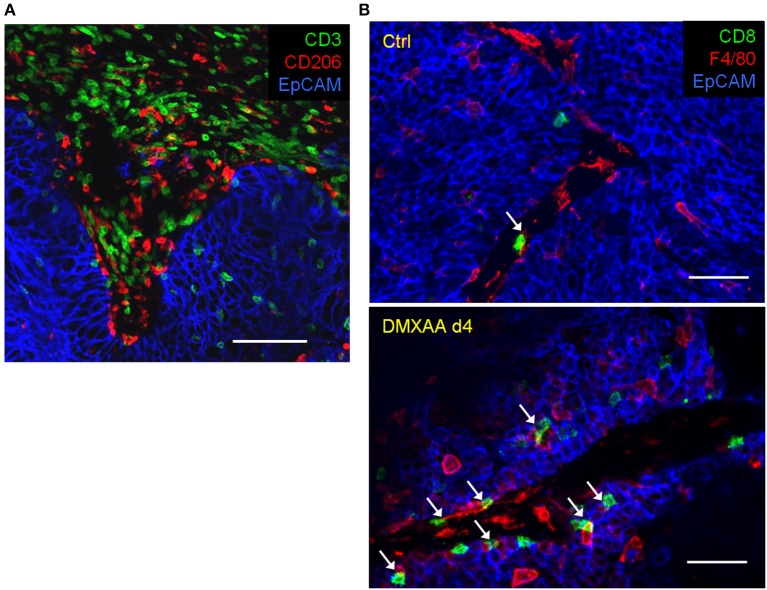
Within tumors, T cells and macrophages are often in contact. **(A)** In a human lung tumor, numerous CD3 T cells are potentially interacting with macrophages (stained by CD206). Bar, 100 μm. **(B)** Interactions between TIL and TAM in mammary carcinoma mouse tumors (MMTV-PyMT) before and after 4 days (4d) of treatment with the STING agonist DMXAA. Of note, the immunotherapeutic agent induces a massive recruitment of CD8 T cells and macrophages (stained by F4/80) with many contacts between both cell types. White arrows indicate TAM-T cells contacts. Bar 50 μm.

Our team has recently shown that, in untreated progressing tumors, TAM have a detrimental impact on TIL ability to migrate within tumors and contact malignant cells (6). By using an experimental system based on thick slices made from fresh tumor biopsies combined with fluorescent imaging microscopy, we evidenced the presence of stable conjugates formed between TAM and CD3 T cells in the stroma of human lung tumors as illustrated in the Figure 2A. If such interactions do not result in T cell activation, macrophages could contribute to sequestering lymphocytes away from tumor cells (6). Remarkably, in mouse mammary tumor models we found that the depletion of TAM with pexidartinib, an inhibitor of the colony stimulating factor 1 receptor (CSF1R), increased the motility of TIL and their ability to reach tumor cells. This is consistent with data obtained in a mouse model of pancreatic carcinoma but with a CD8 T cell-macrophage trapping process that occurs outside the tumor (7). Whether a similar mechanism also affects CD4 T cells is unknown for the moment. In murine lymph nodes, macrophages were shown to sequester γδT cells unable to recirculate in the blood (8).

Altogether, these data suggest that TAM participate to the exclusion of TIL from the vicinity of cancer cells which is considered to be a major hurdle for T cell-based anti-tumor immunotherapy.

### Mechanisms Underlying Blockade of T Cell Migration by TAM

The mechanisms by which TAM prevent CD8 T cells from reaching tumor cells is not known at the moment. We favor an adhesion process between both cell types triggered by an antigen recognition which by itself is insufficient to trigger full T cell activation. This would be in line with data showing antigen-dependent interactions between CD8 T cells and myeloid cells in a spontaneous mammary carcinoma murine model (9). However, the nature of the adhesion molecules involved in such cell-cell conjugates needs to be further investigated.

An effect of TAM on environmental factors controlling the motility of T cells cannot be ruled out. Studies performed over the last few years have provided evidence for a role of the structure of the tissue and the presence of chemokines in regulating the migration of T cells (10). By tracking T cells in fresh human lung tumor slices, we reported an important role of chemokines produced by tumor cells in the ability of T cells to infiltrate tumor islets (11). Such chemokines contribute to a low grade chronic inflammation. In mice harboring mammary tumors, we found that the depletion of TAM resulted in more inflammatory chemokines, such as CCL2 and CXCL10, which are likely to enhance the entry of T cells into the tumor and their intratumoral migration (6). The reason of an enhanced production of chemokines upon TAM depletion is not known for the moment. One possibility is that TAM could participate to the degradation and/or inactivation (e.g., nitration) of inflammatory chemokines, a process reported to occur in murine tumors (12).

A hallmark of advanced tumors is the development of a fibrosis characterized by an excessive accumulation of collagen I, likely to favor tumor progression and prevent antitumor T cell functions by limiting lymphocytes from migrating and contacting tumor cells, as we have previously demonstrated (13). Thus, a dense extracellular matrix (ECM) made by activated carcinoma-associated fibroblasts (CAF) might be responsible for the excluded T cell profile observed in various human carcinomas. The cells and elements that are susceptible to enhance collagen I production by CAF include macrophages. In many physiological situations like breast development, macrophages actively participate to the construction of the tissue (14, 15). In addition, the number of *pro-tissular* macrophages parallels the amount of tissue fibrosis in many human tumors. For example in colorectal tumors and in melanoma, a mesenchymal signature, associated with a bad outcomes and resistance of PD-1 therapy, are characterized by genes involved in extracellular matrix remodeling, angiogenesis, wound healing and TAM suggesting that pro-tissular macrophages and CAF are part of a similar environment (16–18). Evidence obtained in mouse models of colon cancer and pancreatic ductal adenocarcinoma indicates a role of TAM in ECM production within the tumor suggesting that TAM could indirectly inhibit T cell migration through the construction of a dense stroma (19, 20). TAM can fine-tune fibrosis by depositing and/or remodeling the ECM (20) but indirect effects through cross-talks with CAF are also envisioned (Figure 1). In that context, a recent study demonstrates that TAM activate CAF to produce excessive amount of the ECM, excluding T cells from tumor cells, through the secretion of granulin, a growth factor belonging to the epithelin family (21).

### Macrophage-Depletion Strategies May Potentiate Anti-tumor T-Cell Therapies

The aforementioned studies demonstrating a negative impact of TAM on T cells fostered the development of strategies combining pro-tissular macrophage-depletion with approaches that boost T cells. In preclinical mouse tumor models, the depletion of TAM has been combined with T cell-based therapies, both anti-PD-1 and adoptive T cell transfer, which results in enhanced efficacy of the immunotherapy treatment (22–26). For example, we have shown that a macrophage-depletion strategy through CSF1R inhibition, which by itself has a minor effect on the tumor growth, also improved the efficacy of an anti-PD-1 treatment (6).

Based on these results, several therapeutic applications to impair TAM recruitment or survival are either entering or have entered clinical trials (27). CSF1R inhibitors are currently being tested, the most advanced being the small-molecule Pexidartinib (28). However, CSF1R inhibitors have shown very limited antitumor effects in patients as single agents, suggesting the need to combine these inhibitors with other approaches, including immune checkpoint inhibitors. Such combination strategies are ongoing in a number of solid malignancies (NCT02452424, NCT02713529). Macrophages also use the CCL2/CCR2 axis to enter into tumors. Thus, anti-CCR2 approaches are being developed to reduce the number of immunosuppressive macrophages into solid malignancies (29). In addition, chemotherapeutic agents (e.g., gemcitabine, cyclophosphamide, trabectedin), although not specific to TAM, have been shown to deplete myeloid cells (30–32).

## Toward Strategies Favoring an Anti-tumoral Role of TAM

There is increasing evidence that an appropriate activation of macrophages, rather than their depletion, would drastically potentiate an anti-tumor immune response. Macrophages appropriately stimulated by TLR ligands or after abundant cell death, be it induced by radiotherapy, chemotherapy or other means, are key players of an acute inflammation, with numerous consequences. First, inflammatory macrophages release chemokines, leading to the recruitment of innate immune cells and T cells (33). Another major feature of acute inflammation is the activation of the tumor vasculature which controls T cell extravasation (34, 35). In addition, activated macrophages can attack and reduce the density of the intratumoral ECM (36), thus facilitating TIL mobility in the tumor microenvironment. Finally, macrophages are the most abundant cells in tumors, after tumor cells themselves, which constitute a major asset to propagate de novo inflammatory process in the tumor microenvironment. A careful analysis of the various TAM subsets infiltrating human tumors revealed in addition that a high density of anti-tumoral TAM correlated with a favorable prognosis (37).

Historically, various clinical trials were initiated, for instance with the injection of activated macrophages (38) or cytokines and microbial derived molecules (39–43) aiming at activating macrophages directly *in vivo*. Anti-tumoral effects of such macrophage activators have been reported, but such approaches have been globally disappointing. The conclusion to be drawn from this scientific period is that targeting only macrophages cannot induce a systematic tumor regression.

These provisional failures and the very fast increase of T cell knowledge led, in the following years, to an almost complete oversight of anti-tumoral capabilities of macrophages. However, recent advances in myeloid cell biology are putting macrophages back into play, as we will discuss.

### Inhibition of Pro-tumoral TAM Orientation

The inhibition of molecules that contribute to a pro-tumoral TAM orientation represents an interesting strategy. Here we will focus on TGFβ, PI3Kγ, and some HDAC (histone deacetylases) which will nicely illustrate our point.

#### TGFβ Inhibition

The major effect of TGFβ, in large tumors in particular, is to resolve inflammation, to facilitate wound healing and to contribute to immunosuppression. TGFβ does it in various ways. In tumors, TAM are both a source and a target of TGFβ, which is involved in a positive feed-back loop for stabilizing the pro-tumoral TAM phenotype. In parallel, TGFβ promotes the activation of fibroblasts while it inhibits the expression of several molecules necessary for the cytotoxic activity of T cells. Therefore, this molecule appeared to be a central mediator in the TAM/CAF/TIL axis. In the literature on erythropoiesis or on fibrotic diseases, one can find that an anti-TGFβ treatment could be done, in principle, by reducing the concentration of circulating TGFβ with anti-TGFβ antibodies or with TGFβ ligand traps (44), or with pharmacological blockers of TGFβ signaling, such as SB431542 (45). Importantly for the activity of anti-tumor T cells, the combination of TGFβ blockade and anti-PDL1 has been shown to decrease the activity of fibroblasts and the density of ECM relieving the exclusion of T cells from malignant cells (46, 47). Another major effect of TGFβ is the inhibition of IFNβ signaling as we have recently shown in murine spontaneous tumors (48). Type I IFN are central in the initiation of T-cell responses and this finding could be of major importance for combined anti-tumor treatments, in which an anti-TGFβ would not be active on its own, but only for amplifying the potency of T cells. An anti-TGFβ treatment could modify the TAM phenotype and sensitivity to STING agonist within 3 days in this spontaneous tumor model (48).

#### Class II HDAC Inhibition

Class IIa HDAC, which includes HDAC4, act quite differently from other HDAC. A specific Class IIa HDAC inhibitor, which appears to have no effect on T cells, is able to induce an inflammatory state by promoting the infiltration of phagocytic and immunostimulatory CD40^+^ TAM, resulting in an anti-tumoral action (49). Thus, specific inhibitors of class IIa HDAC might prove to be of great interest in combined anti-tumoral therapies. Note that, in different instances, TGFβ effects appear to be mediated by HDAC4 (48, 50, 51). Thus, the two molecules may use common pathways to maintain an immunosuppressive, anti-inflammatory macrophage activity.

#### PI3Kγ Inhibition

PI3Kγ is a PI3K activated by chemokine receptors (52). In myeloid cells, PI3Kγ is important both for the recruitment of monocytes/macrophages and neutrophils (53), and for the resolution of inflammation, in particular with macrophages phagocyting apoptotic neutrophils. In macrophages, the dominant role of PI3Kγ is the resolution of inflammation and immunosuppression (54). Under these conditions, specific inhibitors of PI3Kγ (55, 56) could obviously be of major interest in combined anti-tumoral therapies.

Note that a recent study has evidenced the role of the scavenger receptor clever-1 in TAM pro-tumoral activities. In mouse tumor models, clever-1 blockade leads to macrophage repolarization that become immunostimulatory enhancing T cell responses against tumors (57). A phase I clinical trial with a blocking anti-clever-1 antibody is currently ongoing in various solid tumors (NCT03733990).

### Activation of Anti-tumoral TAM Activities

We would like to shed light on two main pathways promoting an anti-tumoral TAM activity. One involves the CD40 pathway, the other one the induction of type I IFN.

The CD40L-CD40 pathway may be activated by CD40 agonists or with cells that express CD40L as it is the case with activated TIL or NKT (58). In a mouse model of pancreatic cancer, the density of the ECM was shown to be reduced after an anti-CD40 agonist treatment, through the activation of matrix metalloproteinases production by TAM, which may facilitate the motility of T cells (59). Further work from the same group indicates that a CD40 agonist triggers the release of IFNγ and CCL2 responsible for both the recruitment of monocytes/macrophages into the tumor and their polarization toward ECM-degrading cells (60).

Even if anti-CD40 antibodies may not only target TAM and DCs, but also other CD40-expressing cells such as endothelial cells, in combination with gemcitabine, CD40 agonists have already been shown to induce clinical responses in patients with surgically incurable pancreatic cancer (59). This CD40-dependent TAM activation is more efficient when combined with T cell activation (61), or with TLR9 stimulation (62).

Type I IFN has also been shown to enhance anti-tumor activities of myeloid cells. The release of IFNα/β in tumors can be achieved by irradiation (63), some chemotherapeutic agents (64) but more efficiently by a direct activation of the STING (Stimulator of Interferon Genes) molecule. TLR ligands, such as CpG, may also result in the production of IFNα/β by TAM. We have recently shown that this type I IFN contribution to anti-tumoral treatments may be strongly inhibited by TGFβ that accumulates abundantly in spontaneous tumors (48). TGFβ inhibition may therefore be an important element of an efficient combined treatment stimulating anti-tumor activity of TAM.

Overall, the balance toward anti-tumor activity of TAM maybe switched ON if one aims at inhibiting their pro-tissular activity while favoring their pro-immune activity. Various clinical trials are ongoing with such macrophages targeting agents (65).

The duration of an increased anti-tumoral activity of TAM is a question that warrants further investigations. Recruited macrophages with high cytotoxic and phagocytic activities were found to accumulate between 4 and 8 days following treatments (30, 33, 66). Thereafter, factors of the tumor microenvironment, such as VEGF, have been shown to influence the reconstitution of the TAM compartment (30), and to promote tumor outgrowth. The persistence of anti-tumor activity of macrophages may also depend on their interactions with anti-tumor T cells as will be discussed below.

## Activated TAM Cooperate With TIL for a Global Anti-tumoral Activity

An increasing number of reports lead to the conclusion that T cells and TAM can cooperate to fight tumors. We have shown that activated TAM were necessary to reject transplanted tumors after therapeutic vaccination (66) or STING /type I IFN activation (33). Importantly, STING exerts an anti-tumoral activity involving both TAM and T cells (33, 48) with a key role exerted by IFNα/β production by TAM. In such an acute inflammatory context, the depletion of TAM drastically reduced the production of T-cell chemoattractants and the accumulation of CD8 T cells in tumors. Thus, TAM can either favor or prevent intra-tumoral T cell infiltration, depending on whether an inflammatory/immune response has been triggered or not. In addition, a positive feed-back may be observed, with T cells amplifying the activity of immunostimulatory TAM (66). This demonstrates that TAM and anti-tumoral CD8 TIL can work in synergy to reject tumors following an appropriate stimulation.

As a matter of fact, TAM-T cells positive interactions have been observed in various settings, but were rarely put at the forefront. Such positive interactions have been reported after intratumoral injection of TLR3 or TLR9 agonists (67, 68), after the use of checkpoints blockers anti-PD1/anti-CTLA4 (69), or after the adoptive transfer of tumor-infiltrating T cells (70) as well as chimeric antigen receptor (CAR) T cells (71). Table 1 summarizes clinical trials in which a combination of drugs targeting TAM and TIL has been evaluated.

**Table 1:** Ongoing clinical trials targeting TAM and TIL in solid tumors.

	**Macrophage/TIL targets**	**Clinical trial number**	**Investigators**	**Indications**	**Study design**	**Immune response evaluation**	**Phase**
Depletion of pro-tumoral TAM	Anti-CCR2/CCR5/anti-PD1	NCT03184870	Bristol-Myers Squibb	Solid tumors	aCCR2/CCR5 vs. aCCR2/CCR5 + aPD1 vs. aCCR2/CCR5+ chemotherapies	Decrease in regulatory T cells & tumor-associated macrophages	I
	Anti-CFS1R/anti-PD1	NCT02526017	Five Prime Therapeutics, Inc.	Solid tumors	aCSF1R + aPD1 vs. aCSF1R alone	Changes in macrophage and T-cell levels/Changes in gene expression in peripheral T-cell and other leukocyte phenotypes, and levels of peripheral myeloid-derived suppressor cells	I
	Anti-CFS1R/anti-PDL1	NCT03238027	Syndax Pharmaceuticals, Inc.	Solid tumors	aCSF1R alone vs. aCSF1R + aPDL1	Inflammatory cytokines/TIL expansion	I
	Anti-CSF1R/anti-PDL1	NCT02323191	Hoffmann-La Roche	Solid tumors	aCSF1R + aPDL1	TAM depletion	I + II
Inhibition of pro-tumoral TAM activity	Anti-CTLA-4, Anti-PDL1/OX40L Ig	NTC02705482	MedImmune LLC	Advanced solid tumors	OX40L Ig + aPDL1 vs. OX40L Ig + aCTLA4	TIL expansion	I
	Anti-PDL1/OX40L Ig	NTC02221960	MedImmune LLC	Recurrent or Metastatic Solid Tumors	OX40LIg alone vs. OX40L Ig + aPDL1	Biomarkers activity on TIL	I
	PD1-Fc-OX40L	NTC03894618	Shattuck Labs	Solid tumors and lymphomas	1 or 2 inejctions i.t		I
	TGFbRI inhibitor/anti-PDL1	NCT02937272	Eli Lilly and Company	Solid tumors	TGFbRI inh orally alone vs. TGFbRI inh orally + anti-PDL1 i.v		I
	TGFb inhibitor/anti-PD1	NCT02423343	Eli Lilly and Company	Solid tumors (NLSC/HCC)	TGFB inh orally + anti-PD1 i.v		I + II
Activation of anti-tumoral TAM activity	TLR7, 8 agonist/anti-PDL1	NTC02556463	MedImmune LLC	Solid tumors	aTLR7/8 alone vs. aTLR7/8 + aPDL1	TIL expansion/Inflammatory cytokine levels	I
	TRL9 agonist/OX40 agonist	NCT03831295	Stanford Cancer Institute Palo Alto	Solid neoplasms	TLR9 agonist x3 i.t + OX40 agonist x2 i.v and x3 i.t vs. TLR9 agonist x3 i.t + OX40 agonist x3 i.v and x3 i.t		I
	TLR4 agonist/anti-PD1, ICOS agonist, OX40 agonist	NCT03447314	GlaxoSmithKline	Neoplasms	OX40 + TLR4 agonists vs. ICOS + TLR4 agonists vs. aPD1 + TRL4 agonists vs. OX40 + ICOS + TLR4 agonists		I
	STING agonist/anti-PD1	NCT03172936	Novartis Pharmaceuticals	Solid tumors and lymphomas	One vs. 3 doses of STING agonist (i.t) + 1 injection of anti-PD1 (i.v)	Cytokines, TIL expansion in targeted and non-targeted lesions	I
	STING agonist/anti-CTLA4	NCT02675439	Novartis Pharmaceuticals	Solid tumors and lymphomas	3 injections of STING agonist (i.t) vs. 2 injections of STING agonist (i.t) + 1 injection of aCTLA4	Measurement of CD8-TIL counts/RNA expression analysis of IFN gamma and immunomodulatory genes	I
	CD40 agonist/anti-PDL1	NCT02304393	Hoffmann-La Roche	Advanced/ metastatic solid tumors	1 dose of CD40 agonist i.v + aPDL1 vs. 1 dose of CD40 agonist s.c + aPDL1	TIL expansion, PDL1 expression on tumor and immune infiltrating cells	I
	anti-CD47, IFN-α2/anti-PD1, anti-PDL1	NCT02890368	Trillium Therapeutics Inc.	Solid tumors	aCD47 Monotherapy/aCD47 + PD-1/PD-L1 Inhibitor/aCD47 + pegylated IFN-α2/aCD47 + T-Vec/aCD47 + radiation	Anti-tumor activity	I
	GMCSF/iNeo-Vac-P01 (peptides)	NCT03662815	Sir Run Run Shaw Hospital	Solid tumors	iNeo-Vac-P01 (peptides)+ GM-CSF x7 doses	IFN-gamma measurement/CD4 and CD8 T cells subsets	I
	Ad-IFNγ/TIL adoptive transfer	NCT01082887	Nantes University Hospital	Metastatic melanoma	2 injections of Ad-IFNγ (i.t) +2 injections of TIL (i.v)		I+II

### Consequences of TAM-T Cell Cooperation

After facilitating the entry of T cells in sensitized tumors, TAM can interact closely with T cells as illustrated in Figure 2B and present tumor antigens, and thus reactivate them (72). The importance of such a reactivation may be illustrated by the fact that MHC class I expression on tumor infiltrating myeloid cells is strikingly crucial for the rejection of B16 tumor cells by adoptively transferred tumor-specific CD8 T cells (73).

Thus, TAM may help T cells, but reciprocally, T cells can contribute to macrophage activation, and the release of IFNγ seems determinant in this process (66). For instance, the anti-tumor potency of some adoptively transferred T cells was shown to rely on the IFNγ-dependent activation of TAM (74).

Another consequence of such a TAM repolarization is the acquisition of effector functions by these activated cells. Indeed, appropriately activated TAM can phagocyte and engulf tumor cells (75, 76). Additionally, they can kill tumor cells, as shown by several groups (67, 68), including ours (66). TAM, endowed with cytotoxic and cytostatic activities, can kill malignant cells by TNFα secretion (66, 77), NO (74) and sometimes on TRAIL (78).

## Conclusion

The common point of view that TAM are pro-tumoral cells is only correct in one specific situation: that of growing tumors. We have recalled that TAM could play such a role in different ways, including by trapping T cells in the tumor stroma and by reducing their mobility and therefore their capacity to reach cancer cells.

However, we have also discussed that TAM, when appropriately stimulated, have the capacity to cooperate with T cells for an anti-tumoral action. Despite the well-established importance of such a cooperation in anti-infectious immune responses, its importance in anti-tumoral responses has been too often neglected. First, 30 years ago, it has been neglected by those who attempted to treat cancer by only stimulating the innate immune system. More recently, the importance of the TAM-T cell cooperation has again been ignored by those who considered that, for anti-tumoral immune responses, T cells were the good guys and macrophages the bad ones.

We have shown here that an efficient strategy should aim at stimulating both T cells and TAM so as to promote their cooperation. This cooperation is not just about a help provided by TAM to T cells: *the two cell types may be both helpers for the other, and final effectors against the tumor*. Treatments aiming at stimulating only one cell type (only T cells or only macrophages) should be systematically replaced by well-thought combined treatments for stimulating both of them. In particular, *T cell-focused treatments* with checkpoint inhibitors or CAR T cells would greatly benefit being combined with *activators of pro-immune TAM* activities such as CD40 agonists, STING activators or otherof IFNα inducers, and with *inhibitors of pro-tissular TAM functions*, i.e., inhibitors ofTGFβ or class IIa HDAC. The only caveat we would put on it is the potential immune-related adverse effects that may follow efficient tumor rejection. But taking into account that such triple combinations have the greatest chances to promote efficient anti-tumoral therapies would be a fundamental step forward.

## Author Contributions

ED, AT, and NB conceived the article, wrote the first version of the manuscript, and integrated inputs from MG. MG designed the Table 1 with the contribution of NB and the Figure 1 with the contribution of AT. ED and AT designed the Figure 2. All authors approved the final version of the article.

### Conflict of Interest Statement

The authors declare that the research was conducted in the absence of any commercial or financial relationships that could be construed as a potential conflict of interest.

## Abbreviations

CAF: carcinoma-associated fibroblasts
CAR: chimeric antigen receptor
CSF1R: colony stimulating factor 1 receptor
ECM: extracellular matrix
HDAC: histone deacetylase
IFN: interferon
PI3K: phosphatidylinositol 3-kinase
STING: stimulator of interferon genes
TAM: tumor-associated macrophages
TIL: tumor-infiltrating lymphocytes
TGFβ: transforming growth factor beta.
